# Sox9: A potential regulator of cancer stem cells in osteosarcoma

**DOI:** 10.1515/med-2024-0995

**Published:** 2024-07-05

**Authors:** Xiucheng Li, Zuo Lv, Ping Zhou, SongOu Zhang, Chao Jiang

**Affiliations:** Department of Orthopaedics, Shaoxing People’ Hospital, Shaoxing, China; School of Medicine, Ningbo University, Ningbo, China

**Keywords:** Sox9, osteosarcoma, cancer stem cells, regulator, therapeutic target, aggressive

## Abstract

Osteosarcoma is a highly aggressive bone tumor primarily affecting children and adolescents. Despite advancements in treatment modalities, the prognosis for osteosarcoma patients remains poor, emphasizing the need for a deeper understanding of its underlying mechanisms. In recent years, the concept of cancer stem cells (CSCs) has emerged as a crucial factor in tumor initiation, progression, and therapy resistance. These specialized subpopulations of cells possess self-renewal capacity, tumorigenic potential, and contribute to tumor heterogeneity. Sox9, a transcription factor known for its critical role in embryonic development and tissue homeostasis, has been implicated in various malignancies, including osteosarcoma. This review aims to summarize the current knowledge regarding the role of Sox9 in CSCs in osteosarcoma and its potential implications as a prognosis and therapeutic target.

## 1 Introduction

Osteosarcoma is a rare and aggressive type of bone cancer that occurs primarily in children and young adults. It is characterized by the formation of malignant tumors within the bones, typically in the long bones of the body such as the thigh, arm, or pelvis [1]. These tumors can cause significant pain and swelling, and can also lead to the destruction of the surrounding bones and tissue. Osteosarcoma is a highly malignant type of cancer that can spread quickly to other parts of the body, making it one of the most serious types of bone cancer. The cause of osteosarcoma is not fully understood, but it is believed to be a combination of genetic and environmental factors [2]. There is evidence to suggest that genetic mutations, such as those that affect the WWOS and p53 gene, can increase the risk of developing osteosarcoma [3]. Environmental factors such as exposure to radiation or certain chemicals may also play a role [4]. Diagnosis of osteosarcoma typically involves a combination of imaging tests, biopsy, and laboratory tests. Radiographs, such as X-rays or MRI scans, can help to identify the presence of a bone tumor and determine its size and location. A biopsy, which involves removing a small sample of tissue from the tumor, is used to confirm the diagnosis and determine the type of cancer present. Laboratory tests, such as blood tests, can be used to monitor the effectiveness of treatment and check for the spread of the cancer to other parts of the body [5]. Treatment for osteosarcoma typically involves a combination of surgery, chemotherapy, and radiation therapy [6]. Surgery is typically the first line of treatment and involves removing the affected bone and replacing it with a prosthesis. Chemotherapy is used to shrink the tumor and reduce the risk of the cancer spreading to other parts of the body. Radiation therapy can also be used to shrink the tumor and control the spread of the cancer. In recent years, advances in treatment have led to improved outcomes for patients with osteosarcoma. However, the prognosis for patients with this type of cancer can still be poor, and the long-term effects of treatment can be significant. Despite ongoing research and advances in treatment, osteosarcoma remains a challenging and complex disease. It is important for healthcare providers and researchers to continue to work together to better understand the underlying causes of this type of cancer and to develop more effective treatments. This will help to improve outcomes for patients and ensure that they have access to the best possible care and support throughout their journey. Cancer stem cells (CSCs) are a subpopulation of cancer cells that are responsible for the growth, spread, and recurrence of tumors. Unlike other cancer cells, CSCs have the ability to self-renew and differentiate into multiple cell types, allowing them to sustain the growth of tumors over time [7]. This makes CSCs a major contributor to the resistance of cancer to conventional treatments and the ability of cancer to return after treatment has been completed [8]. CSCs are thought to originate from normal stem cells that have acquired mutations that allow them to grow and divide uncontrollably [9]. This can lead to the development of a tumor, which is sustained by the self-renewal properties of CSCs. In order to effectively treat cancer, it is important to target and eliminate CSCs, as they are considered to be the root cause of the disease. Recent advances in research have increased our understanding of CSCs and their role in cancer [10]. This has led to the development of new treatments that specifically target CSCs, including drugs that disrupt their self-renewal abilities or that enhance the body’s immune response to cancer cells. While much more research is needed, the growing body of knowledge about CSCs holds great promise for the future of cancer treatment. CSCs play a critical role in the development, spread, and recurrence of cancer. By understanding and targeting CSCs, we may be able to improve the effectiveness of cancer treatments and improve outcomes for patients. Ongoing research in this area is crucial in order to continue to advance our understanding of this complex disease and to develop new and more effective treatments. Sox9 is a transcription factor that plays a crucial role in the regulation of stem cell differentiation and maintenance [11]. Recent studies have shown that Sox9 is also involved in the development and progression of various types of cancer, including breast, prostate, colon, and pancreatic cancer [12,13]. Sox9 plays a crucial role in the regulation of bone development and the maintenance of normal bone tissue [14]. Recent studies have suggested that Sox9 may also play a role in the development and progression of osteosarcoma [15]. SOX9 was found to be overexpressed in osteosarcoma that were high grade, metastatic, recurrent, or showed a poor response to therapy, suggesting that it may serve as a marker for osteosarcoma [16]. By better understanding the role of Sox9 in osteosarcoma, researchers may be able to develop new and more effective treatments for this type of cancer. Understanding the role of Sox9 in osteosarcoma may lead to a better understanding of the underlying mechanisms of this type of cancer. This knowledge can be used to develop new diagnostic tools and to identify new markers for the early detection of osteosarcoma. Interestingly, recent studies have also highlighted the potential of targeting Sox9 as a therapeutic strategy for treating osteosarcoma. For example, melatonin down regulate the SOX9-mediated signaling pathway, leading to the inhibition of CSCs [17]. Overall, these findings suggest that Sox9 plays an important role in the biology of osteosarcoma stem cells and may represent a promising target for developing new treatments for this devastating disease.

### 1.1 CSCs in osteosarcoma

The concept of CSCs was first proposed in the 1994 by a Canadian scientist named John Dick, who discovered that only a small subpopulation of cells within a leukemia tumor had the ability to initiate and sustain the growth of the cancer [18]. He termed these cells “leukemia stem cells” and suggested that they were similar to normal stem cells in their ability to self-renew and differentiate into multiple cell types. The concept of CSCs gained traction in the early 2000s, when researchers began to apply the concept to solid tumors such as breast cancer [19] and brain cancer [20]. In 2003, Michael Clarke and colleagues at the University of Michigan demonstrated the presence of CSCs in human breast tumors by identifying a subpopulation of cells that expressed high levels of the cell surface marker CD44 and low levels of CD24 [19]. Since then, the existence of CSCs has been confirmed in many other types of cancer, including colon cancer, lung cancer, and osteosarcoma. Researchers have found that CSCs play a critical role in tumor growth, invasion, metastasis, and resistance to traditional cancer treatments such as chemotherapy and radiation therapy. By targeting CSCs, researchers hope to develop new therapies that can more effectively eradicate cancer and prevent recurrence [21].

The first article to report the presence of CSCs in osteosarcoma was published in 2005 by a research team led by Dennis A. Steindler [22]. The results of this study provide evidence for the existence of a small subpopulation of self-renewing bone sarcoma cells that exhibit stem cell-like properties and express activated STAT3, as well as the marker genes of pluripotent embryonic stem (ES) cells, Oct 3/4 and Nanog. These findings support the extension of the cancer stem cell hypothesis to include tumors of mesenchymal lineage and suggest that ES cell homeobox proteins may play a role in non-germ cell tumorigenesis. CSCs have been implicated in the development of treatment resistance and the recurrence of cancer after treatment. Osteosarcoma CSCs can persist even after traditional cancer treatments and are capable of initiating new tumor growth, leading to disease recurrence. The presence of CSCs in osteosarcoma highlights the need for developing new therapies that target CSCs in addition to traditional cancer treatments.

Current approaches for targeting CSCs in osteosarcoma include the use of specific cell surface markers or signaling pathways that are preferentially expressed in CSCs. For example, some researchers have identified CD117 and CD133 as potential markers for CSCs in osteosarcoma. Other researchers have focused on signaling pathways such as the Wnt/β-catenin pathway, which is frequently upregulated in osteosarcoma CSCs.

**Figure 1:**
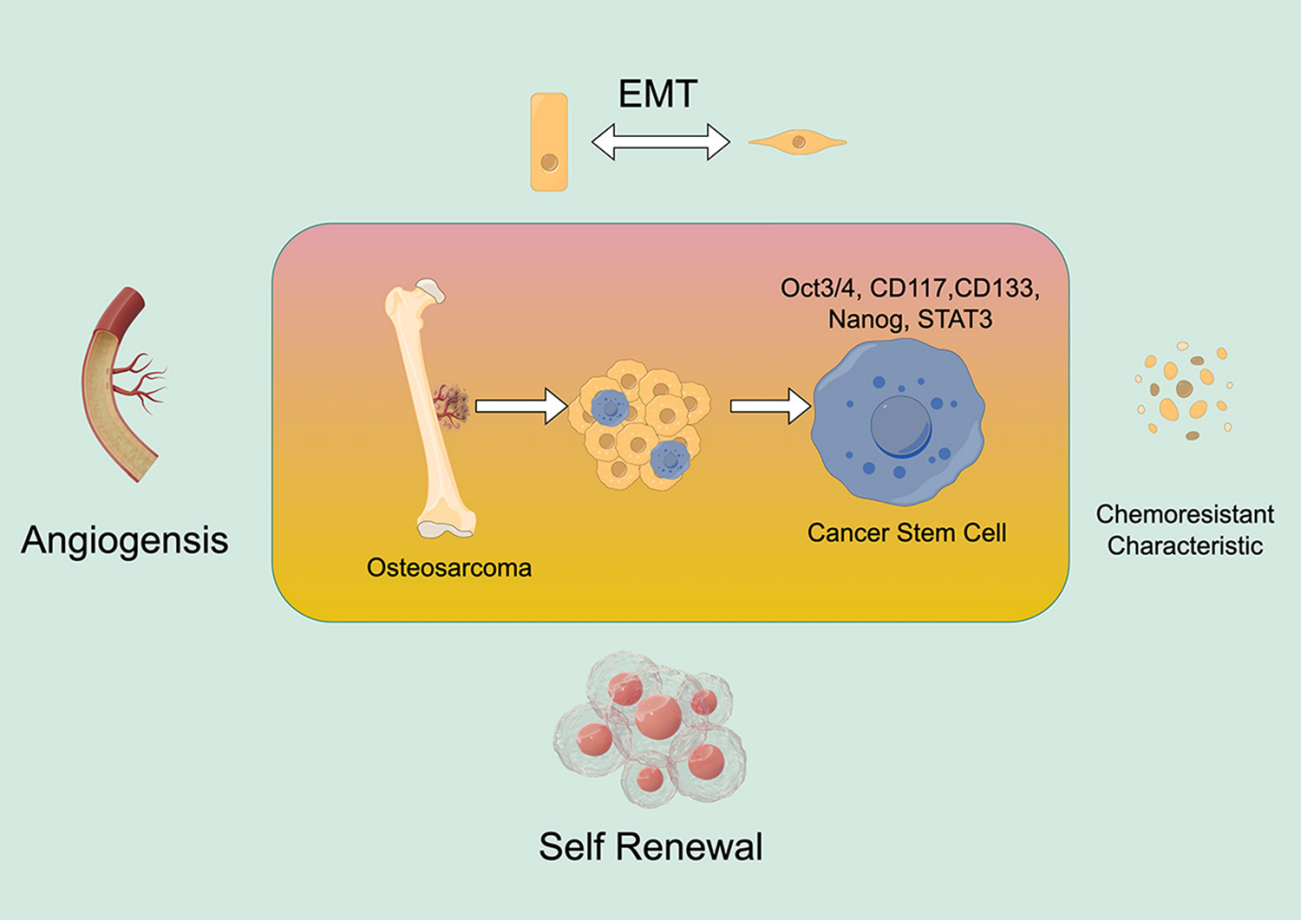
Effects of CSCs in osteosarcoma.

Overall, understanding the role of CSCs in osteosarcoma and developing new therapies that target them is critical for improving treatment outcomes and patient survival in this aggressive cancer. Targeting CSCs in osteosarcoma may provide a way to prevent tumor growth, invasion, and metastasis, and ultimately improve the prognosis and quality of life of osteosarcoma patients (Figure 1).

### 1.2 What is Sox9 and its role in osteosarcoma?

**Figure 2:**
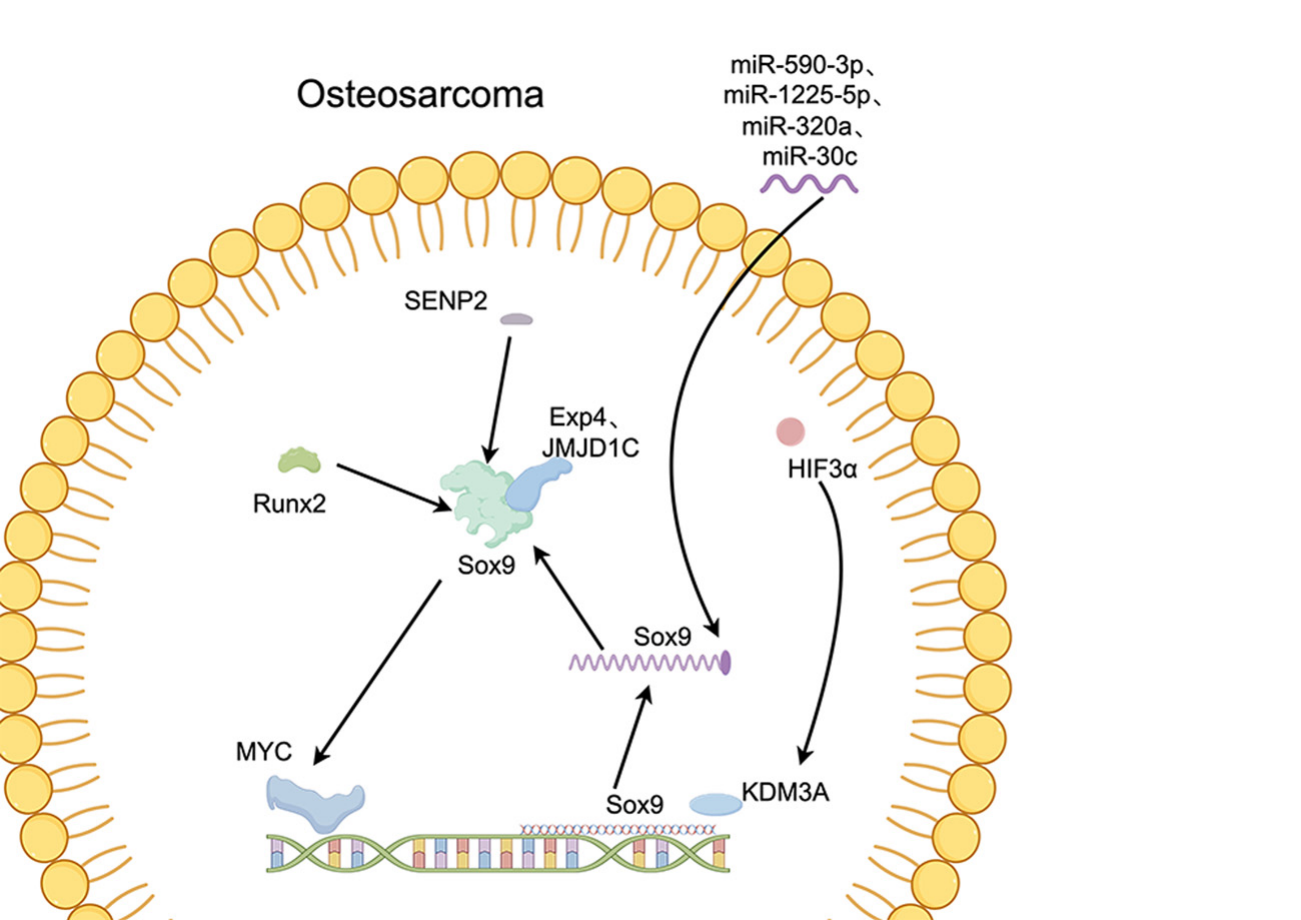
Molecular mechanism of Sox9 in osteosarcoma.

Sox9 is a transcription factor that plays a crucial role in the regulation of gene expression during embryonic development and in the maintenance of normal tissue in adults [23]. It is a member of the SRY-related high mobility group box transcription factor family and is involved in the regulation of a wide range of biological processes, including cell differentiation, cell migration, and cell proliferation [24]. Sox9 was first discovered in 1990 and was initially identified as a gene involved in the development of the testis in mammals. Since then, it has been shown to play a crucial role in the development of several other tissues and organs, including the skeleton, cartilage, and liver [25]. In the skeleton, Sox9 is essential for the proper formation and differentiation of chondrocytes, the cells responsible for the production of cartilage. It regulates the expression of several genes involved in chondrocyte differentiation, including type II collagen, aggrecan, and SOX5 and SOX6 [26]. In the liver, Sox9 is involved in the maintenance of normal liver tissue and in the regulation of the bile ducts [27]. More recently, Sox9 has been implicated in the development and progression of several types of cancer, including osteosarcoma. Studies have shown that Sox9 is over-expressed in cases of dog osteosarcoma, suggesting that it plays a role in the development and progression of this disease [28]. Zhu et al. were the first to report that there is an increase in both Sox9 mRNA and protein levels in human osteosarcoma, and that this upregulation is associated with the advancement of the tumor and poor prognosis in patients [29]. Liu et al. [30] obtained similar results, their study findings indicate that high-grade osteosarcoma tissues exhibit high expression levels of Sox9. Hosseini et al. [16] reported that the high expression of Sox9 in malignant bone tumors was found to be associated with several factors, including large tumor size, high grade, invasive features, tumor recurrence, and poor treatment response. Although Sox9 is related to the prognosis and severity of osteosarcoma, it cannot provide an auxiliary diagnostic role in the pathological differentiation of osteosarcoma and chondrosarcoma [31]. Reduction in Sox9 gene expression leads to decreased proliferation capacity of MG63 cells. In this process, it was discovered that Sox9 can directly target the wnt ligand and its downstream Fzd1 protein in the wnt signaling pathway. These results suggest that Sox9 plays a crucial role in osteosarcoma development and progression by regulating the Wnt1/Fzd1 signaling pathway [30]. In addition to its role in osteosarcoma, Sox9 has also been implicated in the development of several other types of cancer, including liver cancer, prostate cancer, and lung cancer. While the exact mechanisms by which Sox9 contributes to the development of cancer are not yet fully understood, it is thought to play a role in the regulation of several key signaling pathways involved in the regulation of cell growth and survival. miRNA is very popular in the research of osteosarcoma. There are many miRNA targets on the Sox9 sequence. In the study of osteosarcoma, it was found that miR-590-3p [32], miR-1225-5p [33], miR-320a [34], and miR-30c [35] can directly interact with specific binding to Sox9, causing Sox9 degradation or inhibiting its expression. Runx2 is an important transcription factor for osteosarcoma cell survival. Runx2 can directly bind to Sox9, and these two transcription factors can cooperatively activate MYC protein [36]. Sox9 is involved in the effector mechanism of osteosarcoma growth caused by hypoxia. Research shows that HIF-3α can promote the activation of lysine demethylase 3A (KDM3A). KDM3A binds to the gene promoter region of Sox9 and promotes the expression of Sox9, thereby promoting the proliferation and migration of osteosarcoma [37]. Small ubiquitin-like modifier-Specific Protease 2 (SENP2) is a tumor suppressor that also has a good inhibitory effect on osteosarcoma. SENP2 produces osteosarcoma inhibitory effects by directly binding and inhibiting Sox9. Transcription factors need binding partners to jointly regulate gene activation and inactivation [36]. Exp4 [38] and JMJD1C [36] are important binding partners of Sox9 and can inhibit or activate the effects of Sox9 (Figure 2). In conclusion, Sox9 is a transcription factor with a crucial role in the regulation of gene expression and the maintenance of normal tissue. It has been implicated in the development and progression of several types of cancer, including osteosarcoma, and has been shown to play a role in the regulation of key signaling pathways involved in the regulation of cell growth and survival. The ongoing study of Sox9 and its role in cancer holds great promise for the future of cancer research and treatment.

### 1.3 Role of Sox9 in CSCs

**Figure 3:**
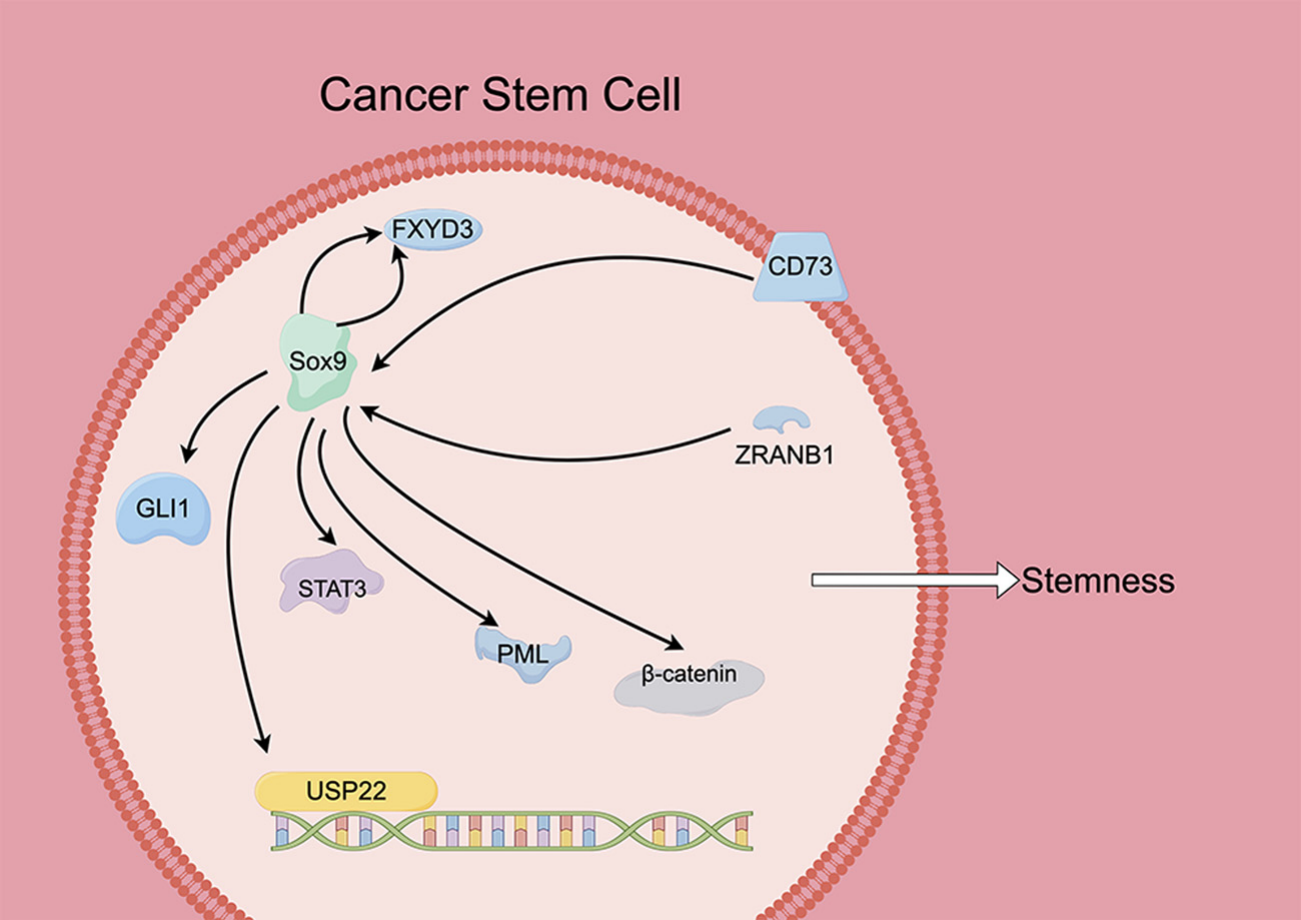
Molecular mechanism of Sox9 in CSCs.

CSCs are believed to be responsible for tumor initiation, progression, and recurrence, as well as for resistance to chemotherapy and radiation therapy. Sox9 has been identified as a marker of CSCs in various types of cancer [13,39], including osteosarcoma [15]. The mechanism of Sox9 in CSCs is complex. For example, there is a significant association between Sox9 and the stemness-regulating enzyme ALDH1A1 [40,41], which is primarily located in the cytoplasm of high-grade ovarian carcinoma with lymph node metastasis. Oh et al. [41] found that silencing of Sox9 led to the complete suppression of cancer stemness properties both *in vitro* and *in vivo*. The interaction between the DD domain of RIPK1 and the transcription activation domain of Sox9 prevents the transcriptional activation of pro-survival and stemness-related genes by the Sox9 transcription factor and block RIPK1-mediated cancer cell death by necroptosis in the cytoplasm, leading to the survival of CSCs in high-grade ovarian carcinoma [41]. In hepatocellular carcinoma, compared to normal adherent growing tumor cells, the sphere model system exhibits a significant enrichment of Sox9 [42]. Thus, Sox9 is an important stem cell factor for sphere formation and proliferation. The promotion of symmetrical cell division by Sox9 regulates self-renewal and tumorigenicity of CSCs [43]. After incomplete radiofrequency ablation, residual hepatocellular carcinoma growth is accelerated by the presence of CSCs. Sox9 was found to promote CD133^+^CSCs proliferation and enhancement of their tumorigenesis ability [44]. Mechanistically, in CSCs, CD73 can activate downstream Sox9 and play a role in maintaining stem cell characteristics [13]. In addition, Sox9 plays a role in glioma stem cells as an upstream target of STAT3 and PML [39]. Tumor necrosis factor receptor-associated factor-binding protein domain (ZRANB1) is a deubiquitinating enzyme. In CSCs, ZRANB1 increases stability by slowing down the ubiquitination of Sox9 [45]. Sox9 can also bind to the promoter of USP22 and promote its expression [45]. Beta-catenin, as a downstream pathway of Sox9, will also be activated [45]. FXYD3 is a critical player in regulating CSC function in ER+ breast cancer. The stem cell-related transcription factor Sox9 promotes FXYD3 expression, which is indispensable for Sox9 nucleus localization, forming a positive feedback loop for FXYD3 amplification and function. FXYD3 interacts with Src and ERα to activate nongenomic estrogen signaling, facilitating ER+ breast CSCs [46]. Deng et al. [47] found that Sox9 directly regulates GLI1, and Sox9-deficient PDA cells have significantly repressed GLI1 levels. Additionally, they identified β-TrCP, a component of the SCF E3 ubiquitin ligase, as a latent SOX9-bound tumor suppressor. Sox9 suppresses the association between β-TrCP and GLI1, promoting GLI1 protein stability and CSC properties. Sox9 is also involved in the lipid metabolism process of CSCs. Sox9 can upregulate ABCA12 lipid transporter to promote tumor stemness and chemotherapy resistance [48] (Figure 3, Table 1).

**Table 1:** A summary table of sox9 in CSCs research

Paper	Cancer type	Model	Drug	Effect	PMID
Li (2019)	Liver cancer	Cell Line: Huh7, HCCLM3	miR-613	miR-613 could inhibit HCC cell dedifferentiation and liver CSCs expansion by targeting SOX9 signaling	PMID: 31075412
		NOD-SCID mice			
Oh (2022)	Ovarian cancer	Cell Line: HGOC	Sox9 shRna	Silencing of SOX9 completely suppressed cancer stemness properties *in vitro* and *in vivo*	PMID: 35159173
		Balb/C mice			
Song (2014)	Esophageal cancer	Cell Line: FLO-1, SKGT-4, BE3, OE33, JHESO, OACP, YES-6, and KATO-TN	Sox9 shRna	shRNA-mediated knockdown of YAP1 or SOX9 in transformed cells attenuates CSC phenotypes *in vitro* and tumorigenicity *in vivo*	PMID: 24906622
		Nude mice			
Olsen (2021)	Small cell lung cancer	Rb1^fl/fl^; Trp53^fl/fl^; Rbl2^fl/fl^; Ascl1^fl/fl^Nude mice	/	ASCL1 promotes neuroendocrine fate and represses the emergence of a SOX9+ nonendodermal stem-like fate that resembles neural crest	PMID: 34016693
Xue (2019)	Breast cancer	Cell Line: MCF-7, BT474	/	SOX9/FXYD3/Src axis in boosting non-genomic estrogen signaling and SOX9 nucleus entry, which is required for maintenance of ER+ 51 breast CSCs and 52 endocrine resistance	PMID: 30206184
		MMTV-PyMT mice			
Domenici (2019)	Breast cancer	Cell Line: MCF-7, T47D, ZR-75	ICI182780	Sox9 acts downstream of Sox2 to control luminal progenitor cell content and is required for expression of the cancer stem cell marker ALDH1A3 and Wnt signaling activity	PMID: 30622340
Ma (2020)	Liver cancer	Cell Line: HCCLM3, Hep3B, MHCC97L, HepG2	MK-2206, SC-79	CD73 plays a critical role in sustaining CSCs traits by upregulating SOX9 expression and enhancing its protein stability	PMID: 32024555
		NOD-SCID mice			

### 1.4 Sox9 as a potential prognosis and therapeutic target for osteosarcoma treatment

Several studies have reported the correlation between Sox9 expression and the clinical outcome of osteosarcoma patients. Studies have shown that Sox9 is overexpressed in osteosarcoma tissues compared to noncancerous bone tissues, and its high expression is associated with advanced clinical stages, positive distant metastasis, and poor response to chemotherapy. Consequently, patients with high Sox9 expression have shorter overall and disease-free survival rates, highlighting the prognostic value of Sox9 in osteosarcoma [16]. Furthermore, Sox9 has been identified as a crucial regulator of osteosarcoma stem cells, contributing to the self-renewal, tumorsphere-forming, and tumor-initiating capacities of these cells [49]. The MAFB-Sox9 reciprocal regulatory axis drives cancer stemness and malignancy in osteosarcoma, providing novel molecular targets that could potentially be therapeutically applicable in clinical settings. Additionally, Sox9 is involved in the regulation of the Wnt1 and Fzd1 signaling pathway, which plays a critical role in osteosarcoma cell proliferation. Inhibition of Sox9 using siRNA has been shown to reduce the expression levels of Wnt1 and Fzd1, resulting in a significant reduction of osteosarcoma cell proliferation [15]. Moreover, melatonin, a hormone with known anticancer properties [50], has been shown to suppress osteosarcoma stem cells by downregulating Sox9-mediated signaling pathways, inhibiting the migration and invasion of osteosarcoma cells, inhibits the sarcosphere formation of osteosarcoma stem cells, and regulates EMT markers in osteosarcoma cells. In an *in vivo* mouse model, melatonin significantly inhibits the initiation and metastasis of osteosarcoma. The study identifies Sox9 as the key transcription factor mediating the effects of melatonin, with melatonin inhibiting CSCs by downregulating the Sox9-mediated signaling pathway in osteosarcoma [17]. Sox9 is an important target for osteosarcoma CSCs. We believe that designing anti-tumor drug delivery systems targeting Sox9 is very promising for the treatment of osteosarcoma. However, there are very few clinical studies on Sox9 as a target. There is only one clinical study registered by a team from Taiwan, China, but the results of the study were not uploaded. As a potential therapeutic and diagnostic target for osteosarcoma stem cells, Sox9 requires more clinical research data. And Sox9, as an important transcription factor, is particularly important for the development and repair of the skeletal system and nervous system. Therefore, when treating Sox9-targeted tumors, these conditions should be considered and its use should be avoided or reduced, especially in patients with damaged skeletal and nervous systems.

## 2 Conclusion

In conclusion, the emerging evidence suggests that Sox9 plays a significant role in the regulation of CSCs in osteosarcoma. By promoting self-renewal, maintenance of stemness, and resistance to therapy, Sox9 contributes to the aggressive behavior and treatment resistance observed in this malignancy. Moreover, Sox9’s involvement in various signaling pathways and its interaction with other transcription factors further highlight its complexity and potential as a therapeutic target. Targeting Sox9 in osteosarcoma CSCs holds promise for developing novel treatment strategies aimed at eradicating the CSC population, reducing tumor heterogeneity, and improving patient outcomes.

However, several challenges and knowledge gaps need to be addressed. Further studies are required to elucidate the precise mechanisms by which Sox9 regulates CSC properties and to identify the downstream effectors involved. Additionally, the development of selective inhibitors or modulators targeting Sox9 activity specifically in CSCs is warranted. There is currently a lack of relevant clinical research, and we need to pay more attention to clinical trials of Sox9. As an important target, Sox9 can be combined with nanomedicine research to form a targeted drug delivery system. Integration of emerging technologies such as single-cell RNA sequencing and functional genomics will provide deeper insights into the heterogeneity and plasticity of osteosarcoma CSCs and their regulation by Sox9.

Overall, understanding the role of Sox9 in osteosarcoma CSCs has significant implications for prognosis prediction, targeted therapy development, and personalized treatment approaches. Further investigations in this field will contribute to the advancement of osteosarcoma research and improve patient outcomes in the future.
